# Compound-specific carbon isotope analysis of short-chain fatty acids from Pine tissues: characterizing paleo-fire residues and plant exudates

**DOI:** 10.1007/s12520-023-01815-3

**Published:** 2023-07-11

**Authors:** Margarita Jambrina-Enríquez, Caterina Rodríguez de Vera, Javier Davara, Antonio V. Herrera-Herrera, Carolina Mallol

**Affiliations:** 1 Departamento de Biología Animal, Edafología y Geología, Facultad de Ciencias, Universidad de La Laguna, San Cristóbal de La Laguna, Tenerife Spain; 2 Archaeological Micromorphology and Biomarker Research Lab (AMBI Lab), Instituto Universitario de Bio-Orgánica Antonio González, Universidad de La Laguna, San Cristóbal de La Laguna, Tenerife Spain; 3 Departamento de Geografía e Historia, Facultad de Humanidades, Universidad de La Laguna, San Cristóbal de La Laguna, Tenerife Spain; 4 ICArEHB - Interdisciplinary Center for Archaeology and the Evolution of Human Behaviour, Universidade Do Algarve, Faro, Portugal

**Keywords:** Organic geochemistry, Lipid biomarkers, Anthropogenic combustion, Organic residue analysis, Degradation of wood, Black layers, Pine biomarkers, Pine resin

## Abstract

Different types of plant tissues and resin can account for the wax lipids found in sedimentary contexts and archaeological samples. Consequently, there is increasing research to characterize the fatty acid carbon isotope ratios of different plant anatomical parts and their plant exudates (resin). With the aim to explore isotopic differences between plant tissues, state of the fine organic matter, effect of thermal degradation, and to identify plant residues we measured the δ^13^C values of short-chain fatty acids (δ^13^C_16:0_ and δ^13^C_18:0_) in: i) dead and fresh (collected and immediately dried) pine needles and branches (*Pinus canariensis*) and pine resin from laboratory-controlled heating experiments and ii) sediment and charred pine tissue samples from a wild pine forest fire. Our results are compared to previously published experimental open-air fire experiments and pine-fuelled archaeological combustion features. We found that for both fatty acid types, there are differences in δ^13^C signatures among anatomical parts and initial moisture content. These data allow us to characterize the isotopic signature of pine tissue and the effect of degradation on isotopic biomarkers, as well as to estimate combustion temperatures in pine-fuelled anthropogenic fires.

**Supplementary Information:**

The online version contains supplementary material available at 10.1007/s12520-023-01815-3.

## Introduction

Paleo-fire residues comprise the by-products of combustion and are dependent on fuel type. In archaeological contexts, combustion residues are commonly charcoal, phytoliths and heated anthropogenic materials such as bone, stone or pottery. Other direct evidence of combustion is comprised by thermally altered sediment, which represents the charred substrate beneath the fire or residual fuel in the case of ash (Aldeias 2017; Mallol et al. 2017). Characterization of fire structures (experimental and archaeological) and their residues by analytical techniques such as compound-specific stable isotope analysis (CSIA) may contribute significantly to our knowledge on past pyrotechnology by providing relevant data on past paleoenvironmental conditions, fuel management strategies, combustion temperatures, the intensity of combustion activity or hearth function, among other aspects.

Simple open-air archaeological combustion structures typically exhibit a stratified sedimentary sequence made up of a reddish-brown layer at the base, overlain by a dark brown/black layer (BL) and capped by a white/yellowish layer (WL) (Mallol et al. 2017). Dark brown/black layers may represent either partially carbonized fuel (wood) (Leroi-Gourhan 1973) or the heated ground over which a fire is built (Mallol et al. 2013), and experimental data indicates that they do not reach combustion temperatures above 350ºC (e.g. Campbell et al. 1995; Mallol et al. 2013; March et al. 2014; Aldeias et al. 2016; Buonasera et al. 2019). These heat altered fire substrates have a high potential for lipid biomarker fingerprint preservation over time (e.g.: Mallol et al. 2013; Jambrina-Enríquez et al. 2019; Leierer et al. 2019; Rodríguez de Vera et al. 2020). In fact, charring processes (incomplete combustion of organic matter under limited oxygen conditions and below 400 ºC) protect organic matter from oxidation and microbial activity preserving their original molecular configuration (Braadbaart and Poole 2008; Wiesenberg et al. 2009; Knicker et al. 2013; Diefendorf et al. 2015; Jambrina-Enríquez et al. 2018).

Lipid biomarkers, molecular-level tracers of organic matter sources, and particularly short-chain fatty acid carbon isotope ratios (δ^13^C_16:0_ and δ^13^C_18:0_), have allowed: i) identification and characterization of different types of animal fats preserved in archaeological combustion structures (March 2013; Choy et al. 2016; Buonasera et al. 2019) and experimental fires (Buonasera et al. 2015), ii) anatomical part discrimination in fresh and charred plants heated under controlled laboratory conditions, experimental and archaeological fires (e.g. Jambrina-Enríquez et al. 2019) and iii) assessment of the thermal degradation effect on lipid biomarker composition of fresh dried plant biomass from controlled laboratory heating sequences (Knicker et al. 2013; Diefendorf et al. 2015; Jambrina-Enríquez et al. 2019; Tomé et al. 2022). Regarding plant biomass, to identify the source of organic residues encrusted on pottery or present in combustion structure sediment samples, the current plant oil δ^13^C_16:0_ and δ^13^C_18:0_ reference database focuses on fresh seed oils (Woodbury et al. 1998; Spangenberg and Ogrinc 2001; Steele et al. 2010) and fresh C3-leaf oils (Chikaraishi et al. 2004a, b) and only a few studies incorporate fresh and charred wood oils (e.g.: Jambrina-Enríquez et al. 2018; Tomé et al. 2022).

Characterizing fatty acid carbon isotope signatures in fresh and dead leaves/needles and branches, and the isotopic changes that occur upon heating and burning are important challenges for the identification of burned plant residues in archaeological black layers and for the determination of the state of the fine organic matter associated with fire substrates (fresh, dead and rotten leaves) and with woody fuel (fresh, dead or rotten wood). Over the past years, experimental charcoal analysis has focused on the study of taxonomic preferences in fuel selection, the calibre or the state of the wood, and comparisons with the anthracological record in archaeological combustion contexts as informative of firewood management, firewood selection criteria and use by prehistoric societies (Théry-Parisot et al. 2010; Théry-Parisot and Henry 2012; Henry and Théry-Parisot 2014). Observation and quantification of anatomical alteration in wood on experimental charcoal samples using macroscopic and microscopic approaches indicate an equal response to fungal attacks by different species of pine (*Pinus canariensis* and *Pinus sylvestris*) (Vidal-Matutano et al. 2021). These studies also discriminate between fresh, dead, or rotten wood in charcoal samples from archaeological ash layers from fire pits and flat hearths although with some differences from the experimental dataset linked to the effects of post-depositional processes or flotation and wet sieving (e.g., Vidal-Matutano et al. 2017, 2021; Allué and Mas 2020; Liu et al. 2022). A biomarker approach to different states of pine residues in combustion contexts could contribute a new, complementary proxy to paleofire research.

Although the effect of heat on pine lipid biomarkers is little known (e.g. Oros and Simoneit 2001; Diefendorf et al. 2015), the lipid (McKern 1965; Oros and Simoneit 2001; Ustun et al. 2006; Otto et al. 2007) and isotopic composition (e.g., Diefendorf et al. 2010) of pine is well documented and homogeneous across species, which implies a possibly similar effect of heat on different taxa. For example, different pine species shows similar carbon stable isotope values in their bulk (δ^13^C_org_) and individual organic compounds (δ^13^C_n-alkanes_) (Diefendorf et al. 2010). They all fall within the range of C3 plants because conifers fractionate similarly during photosynthesis (e.g., Diefendorf et al. 2010). Also, the monoterpene composition shows that the chemosystematics of the subgenus *Pinus* (*P. halepensis*, *P. brutia*, *P. nigra*, *P. pinea* and *P. canariensis*)*,* specifically, which is dependent upon the plant's genotype (McKern 1965), is the same for *P*. *canariensis*, *P. halapensis*, *P*. *nigra* (Roussis et al. 1995) and *P*. *sylvestris* (Ustun et al. 2006).

In the Canary Islands (Spain), *P. canariensis*, an endemic pine with similar volatile constituents as other documented pine species (Roussis et al. 1995; Ustun et al. 2006; Otto et al. 2007), and with an outstanding resistance to fire and a heartwood with an extraordinarily high resin content (pitch wood), dominates the islands’ forests (Climent et al. 1998). Canarian pine resin is known to have been heavily used for tar production in the fifteenth-sixteenth centuries AD, during the times of early European colonization and commercial trade with the Americas, which demanded large amounts of tar to waterproof boats (caulking), as well as to waterproof building rooftops (Brito and Rodríguez 2008). The aboriginal prehispanic population may have also used *P. canariensis* resin in bandages for medical purposes (bandages) (Chil Naranjo 1990). So far, biomarker approaches focused on C isotope composition have not been applied in any archaeological context, including Canarian early European or indigenous archaeological contexts, to explore resin use.

Here, we present the results of a series of experiments (i.e., controlled laboratory heating sequences) using pine (*Pinus canariensis*) (fresh and dead (collected and immediately dried) needles and branches) and charred plant samples and sediment from a recent pine forest wildfire in Tenerife, Canary Islands (2018). For both cases, we analyzed the charred organic residues through a lipid biomarker approach focusing on compound-specific stable isotope ratios, specifically short-fatty acid carbon isotopic ratios. Our goals are i) to explore the potential of discriminating between fresh and dead pine branches and needles through their carbon isotopic signatures on individual short *n*-fatty acids, ii) to identify potential sources of the organic charred residues found in archaeological black layers, i.e., charred fuel (branches) vs. charred soil cover (mainly leaves) and iii) to characterize the C isotopic signatures of pine resin.

Finally, to test our results, and since the isotopic similarity among pine species (Diefendorf et al. 2010, 2015) facilitates the comparison of our experimental δ^13^C_16:0_ and δ^13^C_18:0_ dataset with δ^13^C_16:0_ and δ^13^C_18:0_ values of combustion features fuelled with wood from different pine species, we compared our experimental laboratory data and wildfire data with existing δ^13^C values of short-chain fatty acids from: i) an experimental open-air fire fuelled with *P. canariensis* (Buonasera et al. 2019), ii) Canarian indigenous archaeological combustion zone tramples (Roques de García Rockshelter, Tenerife, Canary Island, Spain; Tomé et al. 2022) and iii) the black layers of archaeological combustion structures from two Middle Paleolithic sites fuelled with pine (El Salt, Spain and Crvena Stijena, Montenegro, Jambrina-Enríquez et al. 2019).

## Samples and methods

### Samples

#### Reference collection and pine forest wildfire

*P. canariensis* branches, needles, and plant exudates (resin) for laboratory-controlled heating experiments were collected in the Corona Forestal Nature Park in Tenerife (Canary Islands, Spain), a very rich pine forest formation dominated*.* In the field, fresh branches (3 cm diameter) and needles were cut directly from the trees (2 m above the ground), fallen branches (3 cm diameter, bark free) and needles were collected from the ground, and resin exudates oozing out of the trunk were collected in situ. At the site three individuals of *P. canariensis* were sampled and for each tree, 20–50 fresh and 20–50 dried needles and 2 fresh branches and 2 dried branches were randomly sampled and stored in paper bags.

**Fig. 1:**
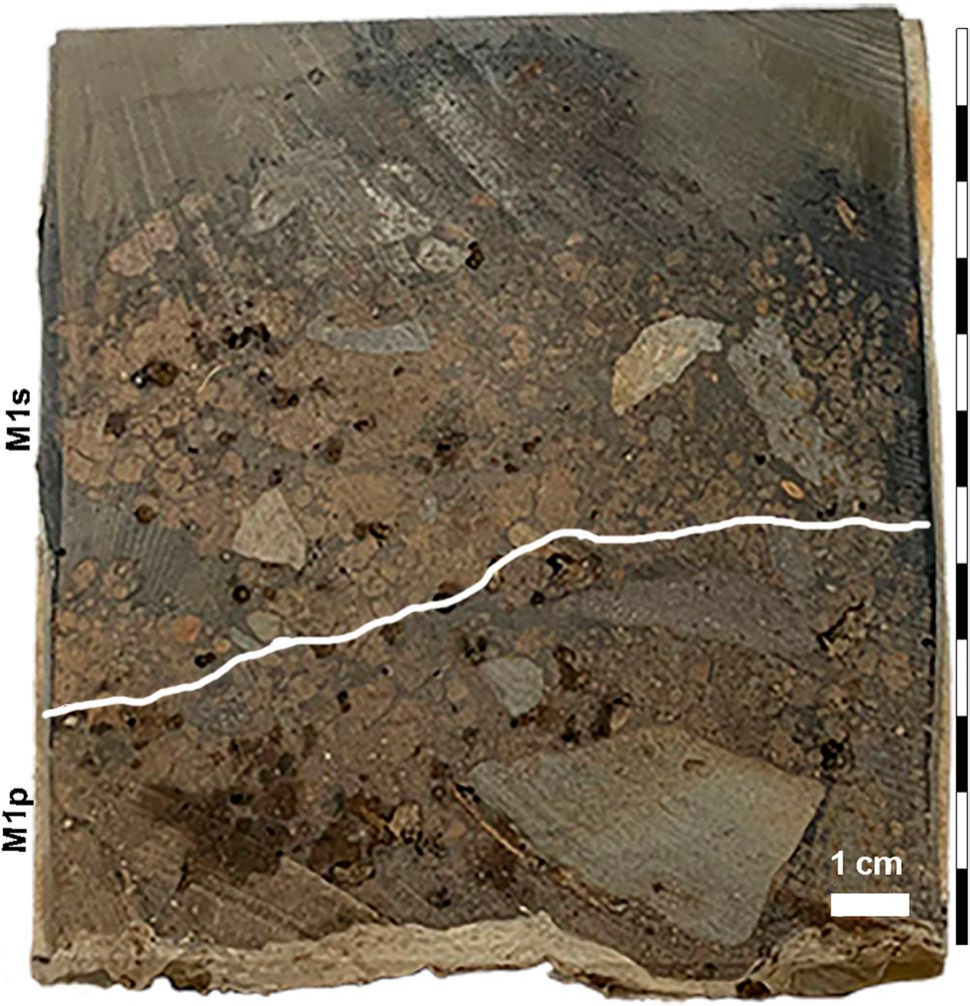
Sedimentary profile (M1) from the pine forest wildfire

Samples of *P. canariensis* branches and needles were collected at the Corona Forestal Natural Park (Tenerife) one month after a four-day-long wildfire burnt down approximately 350 hectares of pine forest and scrubland. At the sampling location, within the perimeter of the fire-affected area and labelled as site M1 (N 28º11′06″, W 16º35′58″, 1678 m asl), we collected branch ash, charred bark-free branches with an unburned inner section, and charred needles from the surface, along with an intact sediment sample collected using a 20 cm-piece of PVC pipe (15-cm-diameter). At the lab, we subsampled a 0–5 cm segment (M1s) and a 5–10 cm segment (M1p) for lipid analysis. The sediment samples are light brown, loose, silty, and organic-rich, without any apparent thermal alteration (i.e., rubefaction) except for the presence of charred needles, charred bark-free branches, and branch ash at the top (Fig. 1).

All the samples were collected using nitrile gloves, sterilized metal tools (for sediment samples), and packed in aluminum foil. Sediment samples were stored at -20 °C to prevent bacterial degradation. Fresh and dead plant tissues were rinsed with distilled water to eliminate any extraneous materials and oven-dried at 60 °C for 24 h; sediment samples were only dried at 60 °C over 48 h (Gamarra and Kahmen 2015; Jambrina-Enríquez et al. 2018).

#### Samples from open-fire experiments and archaeological combustions structures

The experimental open-air fire made by Buonasera et al. (2019) was fuelled with mostly bark-free *P. canariensis* wood (5 kg of dried and pre-cut wood from a local firewood business) over a crushed, compacted basalt sediment. The authors reported δ^13^C_16:0_ and δ^13^C_18:0_ values from the white layer (wood ash with some charcoal) and black layer (basalt sediment substrate), along with unheated wood fragments as control samples.

At Los Roques de García (Tenerife, Canary Islands, Spain), a archaeological highland rockshelter occupied in prehispanic times, Tomé et al. (2022) analyzed the micromorphological, molecular and isotopic fingerprints a 50 cm-thick stratified profile including two layers formed by human trampling on a surface with fire residues: MFU8 (the oldest occupation surface at the site) and MFU19.

Middle Paleolithic combustion structure H50 from El Salt (Spain), was studied through anthracology (Vidal-Matutano et al. 2017), micromorphology (Leierer et al. 2019) and biomarker (Leierer et al. 2019; Jambrina-Enríquez et al. 2019) approaches. H50 is in the outer cluster of the Unit Xb archaeological assemblage (Unit X: ~ 50 ka BP, Galván et al. 2014), and comprises a circular (90 cm-diameter), bowl-shaped depression filled with dark, greasy sediment (black layer) and cobble-shaped imprints on its surface. The imprints are filled with ashy sediment (white layer) (Leierer et al. 2019). The white layer contains *Pinus nigra-sylvestris* charcoal fragments in low and medium biodegradation states pointing to seasoned green and dead wood (Vidal-Matutano et al. 2017). The concentrations of short-chain fatty acids in the ash layer were below the needed concentration to obtain reliable δ^13^C values and only the black layers (BL1, BL3 and BL4) reported sufficient C_16:0_ and C_18:0_ concentrations to yield δ^13^C_16:0_ and δ^13^C_18:0_ values (Jambrina-Enríquez et al. 2019).

Finally, we selected three BLs (BL-8, BL-10 and BL-11) with δ^13^C_16:0_ and δ^13^C_18:0_ values (Jambrina-Enríquez et al. 2019) from stratigraphic Unit XXIV at the Middle Palaeolithic site of Crvena Stijena (Montenegro). This unit is a 2.3 m-thick layer composed of multiple, stacked combustion features extremely rich in charcoal (*Pinus sylvestris,* Shaw 2017), wood ash, burnt and unburnt bone fragments and dated to between 52.2 and 78.3 Ka (Morley 2017).

### Methods

#### Laboratory heating sequences

Prior to charring, branches (*n* = 2) and needles (*n* = 20) from each pine individual were cut into small pieces (3–4 cm thick and 1 cm long, respectively) and weighted (average of fresh and dead needles: between 2 g for untreated and 4 g for 450 ºC; average of fresh and dead branches: between 8 g for untreated and 22 g for 450 ºC). For resin we used between 2 g (untreated) and 8 g (450 ºC). Although experimental data in combustion structures indicates combustion temperatures below 350ºC (Campbell et al. 1995; Mallol et al. 2013; March et al. 2014; Aldeias et al. 2016; Buonasera et al. 2019) we included laboratory heating sequences up to 450ºC to assess the final ^13^C enrichment phase related to the preferential loss of ^12^C-enriched CO_2_ from organic matter upon heating, previously reported in long-chain *n*-alkane δ^13^C values from fresh and charred leaves by Diefendorf et al. (2015) and Jambrina-Enríquez et al. (2018). All the samples were heated in ceramic crucibles (4.2 cm diameter × 2.4 cm height) wrapped with Al foil to limit oxidation during charring extent; the covers were not air-tight (e.g., Wiesenberg et al. 2009; Wiedemeier et al. 2015; Jambrina-Enríquez et al. 2018). A muffle furnace was used to heat the samples (needles, resin and branches from the same fresh pieces) for 1 h at 150, 250, 350 and 450 ºC with a ramp rate of 26 ºC/min (Kuo et al. 2008). After heating, the products were cooled down in the closed furnace, the charred branches and needles were subsequently ground to a fine powder before lipid extraction and the resin was directly put on the lipid extraction vials.

#### Lipid extraction and fatty acids gas chromatography-mass spectrometry (GC–MS) analysis

Lipid extraction and GC–MS analysis were conducted following the protocol described by Jambrina-Enríquez et al. (2018). Lipids were extracted from unburnt and burnt tissues (branches and needles), and resin with dichloromethane/methanol (DCM:MeOH, 9:1, v:v) by ultrasonic extraction (3 × 30 min) followed by centrifugation (3 × 10 min at 4700 rpm) and filtered through annealed glass wool and GF/F grade glass microfiber filter.

The total lipid extract was separated into five different polarity fractions through a silica gel column (filled with glass wool, 0.1 g pure quartz sand and 1 g of activated silica) using several mobile phases. Fraction 1 (*n*-alkanes) was eluted with 3/8 of column dead volume (DV) with hexane, fraction 2 (aromatics) with 2DV in hexane:DCM (8:2, v/v), fraction 3 (ketones) with 2DV in DCM, fraction 4 (alcohols) with 2DV in DCM/ethyl acetate (EtOAc) (1:1, v/v), and fraction 5 (fatty acids) with 2DV in EtOAc. All the fractions were dried under a N_2_ flow. Here we only center on fatty acid fraction since this study is focused on compound-specific carbon isotope analysis of short-chain fatty acids. Once added 1 μL of methyl nonadecanoate (400 mg/L in DCM) as internal standard (IS), the fatty acid fraction was derivatised to their respective methyl esters (FAMEs) by adding to the extract 5 mL MeOH, 400 μL of H_2_SO_4_ and heating them at 70 ˚C for 240 min. Thereafter, the solution was neutralised with 10 mL of saturated bicarbonate solution and FAMEs were extracted with hexane (3 × 3 mL), dried under N_2_ flow and reconstituted with 50 μL of DCM (Jambrina-Enríquez et al. 2019; Rodríguez de Vera et al. 2020).

FAMEs were analysed twice by gas chromatography with a coupled mass-selective detector (GC-Agilent 7890B, MSD Agilent 5977A) equipped with an HP-5MS capillary column (30 m length × 0.25mmi.d., 0.25 mm film thickness). The GC was programmed to an initial temperature of 70 ºC for 2 min, then heated with a heating rate of 12 ºC/min to 140 ºC and to final temperature of 320 ºC with a heating rate of 3 ºC/min and held for 15 min, with helium as the carrier gas (1 mL/min). The multimode injector was held at a split ratio of 5:1 at an initial temperature of 70 ºC for 0.85 min and heated to 300 ºC at a programmed rate of 720 ºC/min.

Palmitic acid (C_16:0_) and stearic acid (C_18:0_) methyl esters were identified by comparison of their retention times and mass spectra with those of reference compounds (37 component FAME mix C_4_-C_24_, 200–600 mg/L in DCM).

#### Compound-specific carbon isotope analysis of C_16:0_ and C_18:0_ fatty acids

Carbon stable isotope values of individual C_16:0_ and C_18:0_ fatty acids were determined by a gas chromatography-combustion-isotope ratio mass spectrometry (GC-C-IRMS) system consisting of a Thermo Scientific Isotope Ratio Mass Spectrometer Delta V Advantage coupled to a GC Trace1310 through a Conflo IV interface with a temperature converter GC Isolink II. Injection was performed for those samples whose reconstituted extract had a concentration above the GC-C-IRMS limit of quantification (5 mg/L). The analysis was conducted under the conditions described by Jambrina-Enríquez et al. (2019). The chromatograph used a Trace Gold 5-MS (Thermo Scientific) capillary column (30 m length, 0.25 mm i.d. and 025 mm phase thickness), and Helium as carrier gas (1.2 mL/min). The temperature programme comprised a 2 min isothermal period at 70 ºC, followed by an increase to 140 ºC at a rate of 12 ºC/min and held for 2 min. Then, the temperature increased to 320 ºC at a rate of 3 ºC/min and held for 15 min while the combustion reactor temperature was maintained at 1000 ºC. Samples were injected (1 µL) in splitless mode using a Programmed Temperature Vaporising (PTV) injector programmed with an evaporation step with the temperature increasing from 60 ºC to 79 ºC (held 30 s, rate 10 ºC/min), followed by a transfer stage increasing to 325 ºC (held 3 min, rate 10 ºC/s) and a cleaning step with temperature increasing to 350 ºC.

A FAME standard mixture F8-3 (C_14:0_-C_20:0_, Arndt Schimmelmann Biogeochemical Laboratories, Indiana University) of known isotopic value was run prior to each batch of analyses. Each sample was measured three times and resulting standard deviations were ≤ 0.6‰. Both standard mixture and samples were run in triplicate. The data are reported in the “Delta” notation as ‰ relative to Vienna Pee Dee Belemnite (VPDB). Finally, the isotopic signature of the methyl groups introduced in the free fatty acid results were corrected using the mass balance equation used by Goodman and Brenna (1992).

#### Statistical analysis

Statistical analysis was applied to the δ^13^C values to validate or refute our results. First, a normality test was used to check the distribution of the data. The resulting data did not follow a normal distribution. As a result, we decided to apply a Pearson correlation Test and two types of non-parametric tests: Mann–Whitney and comparison between related samples using a bilateral test and the Wilcoxon index, both at 95% confidence. We also applied a correlation index to test the relation between temperature and changes in the δ^13^C_16:0_ and δ^13^C_18:0_ values for fresh and dead branches and needles. The statistical analysis was performed using XLSTAT v. 2020.2.2 software.

## Results

### Laboratory heating sequences

Fresh and dead needles charred up to 250 ºC, as well as reference samples dried at 60 ºC had lower δ^13^C_16:0_ and δ^13^C_18:0_ values (δ^13^C_16:0_ ranges from − 38.1 to − 33.4‰, and δ^13^C_18:0_ ranges from =  − 35.0 to − 31.5‰) compared with fresh and dead needles charred at 350 and 450 ºC (δ^13^C_16:0_ ranges from − 35.1 to − 25.6‰, and δ^13^C_18:0_ ranges from =  − 28.0 to − 24.5‰). Fresh branches (burnt and unburnt samples) had lower δ^13^C_16:0_ and δ^13^C_18:0_ values (δ^13^C_16:0_ ranges from − 32.2 to − 30.4‰, and δ^13^C_18:0_ ranges from =  − 29.6 to − 27.4‰) compared with dead branches (burnt and unburnt samples) (δ^13^C_16:0_ ranges from − 27.1 to − 22.6‰, and δ^13^C_18:0_ ranges from =  − 27.1 to − 21.5‰), with the exception of the fresh branches samples charred at 450 ºC, which reported similar values than dead branches samples (δ^13^C_16:0_ =  − 26.4‰, and δ^13^C_18:0_ =  − 25.2‰). The resin burnt at 450 ºC did not produce residues so lipid composition and compound-specific isotope analysis on individual fatty acids was not performed. Higher δ^13^C_16:0_ and δ^13^C_18:0_ values were reported on unburnt and burnt samples at 150 ºC (δ^13^C_16:0_ ranges from − 26.8 to − 25.8‰, and δ^13^C_18:0_ ranges from =  − 26.7 to − 25.1‰) and lower δ^13^C_16:0_ and δ^13^C_18:0_ values on charred samples at 250 and 350 ºC (δ^13^C_16:0_ ~  − 27‰ and δ^13^C_18:0_ ~  − 27‰) (Table 1).

**Table 1:** Carbon isotope values of individual *n*-fatty acids in fresh, dead and experimentally heated *P. Canariensis* needles and branches, along with charred needles, charred bark-free branches and wood ash from the *P. canariensis* wildfire. Data without Suess effect correction are presented using the δ^13^C notation (normalized to the VPDB standard) ± 1 standard deviation (σ). nd = non determined

Sample ID			δ^**13**^C_16:0_ (‰)	σ	δ^**13**^C_18:0_ (‰)	σ
Pine tissues	Fresh needles	Unheated	− 38.1	± 0.4	− 35.0	± 0.2
		150 ºC	− 37.9	± 0.3	− 33.3	± 0.1
		250 ºC	− 37.4	± 0.3	− 33.1	± 0.2
		350 ºC	− 31.5	± 0.0	− 24.5	± 0.1
		450 ºC	− 26.0	± 0.4	− 26.7	± 0.4
	Dead needles	Unheated	− 36.4	± 0.4	− 33.8	± 0.4
		150 ºC	− 35.2	± 0.2	− 35.3	± 0.1
		250 ºC	− 33.4	± 0.1	− 31.5	± 0.2
		350 ºC	− 28.8	± 0.1	− 27.1	± 0.1
		450 ºC	− 25.6	± 0.1	− 25.7	± 0.2
	Fresh branches	Unheated	− 30.7	± 0.1	− 28.0	± 0.2
		150 ºC	− 32.2	± 0.2	− 29.6	± 0.3
		250 ºC	− 31.3	± 0.1	− 29.2	± 0.3
		350 ºC	− 30.4	± 0.4	− 27.4	± 0.2
		450 ºC	− 26.4	± 0.4	− 25.2	± 0.6
	Dead branches	Unheated	− 27.1	± 0.2	− 21.5	± 0.3
		150 ºC	− 26.6	± 0.3	− 27.1	± 0.3
		250 ºC	− 27.0	± 0.4	− 26.2	± 0.4
		350 ºC	− 22.6	± 0.4	− 25.0	± 0.6
		450 ºC	− 25.8	± 0.1	− 26.0	± 0.1
	Resin	Unheated	− 25.8	± 0.3	− 25.1	± 0.4
		150 ºC	− 26.8	± 0.2	− 26.7	± 0.1
		250 ºC	− 27.3	± 0.3	− 26.9	± 0.4
		350 ºC	− 27.5	± 0.4	− 27.0	± 0.6
		450 ºC	nd	nd	nd	nd
Pine Wildfire	Charred bark-free branches		− 29.3	± 0.5	− 28.2	± 0.4
	Charred needles		− 23.9	± 0.4	− 25.7	± 0.4
	Branch ash		− 27.8	± 0.4	− 26.8	± 0.2
	M1s (sediment)		− 30.7	± 0.5	− 26.3	± 0.2
	M1p (sediment)		− 30.7	± 0.3	− 25.8	± 0.4

### *Pinus canariensis* wildfire

Charred needles (δ^13^C_16:0_ =  − 23.9 ‰ and δ^13^C_18:0_ =  − 25.7‰) collected from the surface at site M1 showed higher δ^13^C_16:0_ and δ^13^C_18:0_ ranges than charred bark-free branches (δ^13^C_16:0_ =  − 29.3‰ and δ^13^C_18:0_ =  − 28.2‰) and wood ash δ^13^C_16:0_ =  − 27.8‰ and δ^13^C_18:0_ =  − 28.2‰). The sediment sample yielded δ^13^C_16:0_ values ~  − 31‰ and δ^13^C_18:0_ values ~  − 26‰ (Table 1).

## Discussion

**Fig. 2:**
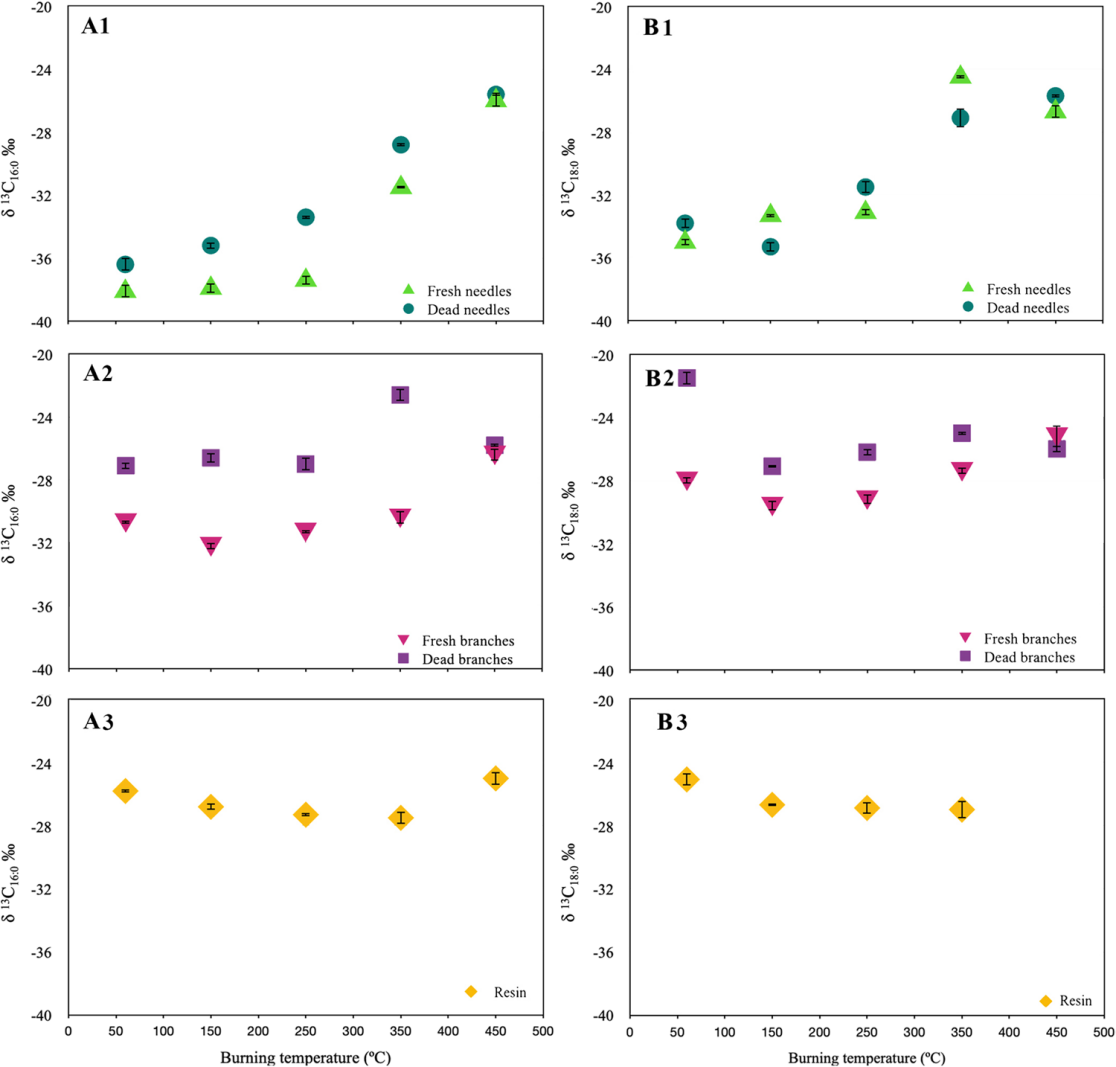
Effect of thermal degradation on δ^13^C_16:0_ (**A**_1-3_) and δ^13^C_18:0_ (**B**_1-3_) values for fresh and dead *Pinus canariensis* tissue (1: needles, 2: branches, 3: resin)

Isotopic variations were identified among the heated fresh and dead pine tissues with increasing combustion temperature (see Online Resource 1, ESM-1, for statistical test data). As temperature increased needles and branches were enriched in ^13^C up to 450° C (Pearson’s correlation coefficient ≥ 0.7), except for charred dead branch samples (Pearson’s correlation coefficient ≤ 0.5) for which the effect of thermal alteration was minor (Fig. 2AB1-2 and ESM-1). Under smouldering conditions (≤ 250 ºC) the difference in the δ^13^C_16:0_ and δ^13^C_18:0_ values of needles are on average − 0.5 ‰ ± 0.2 and about − 1.3‰ ± 0.9, respectively with a shift to higher δ^13^C values at 350–450 ºC (~ 4–5‰). Dead needles (unburnt and burnt up to 350 ºC) are enriched by ~ 3‰ relative to the fresh needles. This enrichment is more evident in *n*C_16:0_. However, at 450 ºC, fresh and dead needles recorded similar values regardless of the state of the degradation (Fig. 2AB1).

**Fig. 3:**
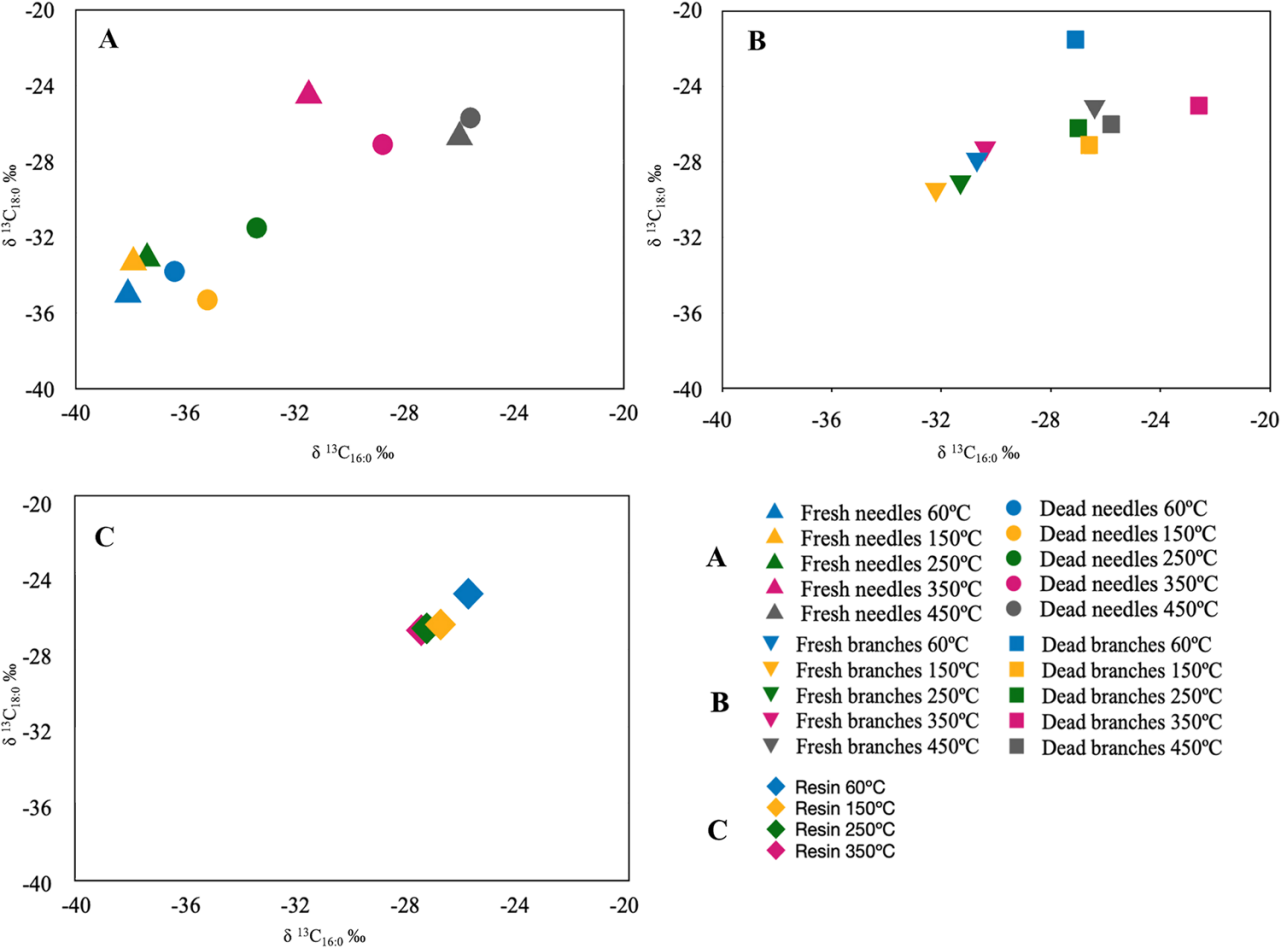
δ^13^C_16:0_ and δ^13^C_18:0_ values of plant tissues from laboratory-controlled heating experiments in fresh and dead needles (**A**), fresh and dead branches (**B**) and resin (**C**)

Our findings on the experimentally heated fresh needles agree with previous studies on fresh leaves, showing that thermal degradation by charring at smouldering temperatures does not affect the short-chain fatty acid C isotope signatures, and that at higher temperatures, there is isotopic enrichment of up to 5‰ relative to the unburned tissue (Ballentine et al. 1998; Jambrina-Enríquez et al. 2019). Plots of the δ^13^C_16:0_ and δ^13^C_18:0_ values of fresh and dead needles under smouldering conditions (≤ 250 ºC) fall together and in a different area than the charred samples heated at 350–450 ºC, indicating that the state of degradation in needle samples cannot be discerned (Fig. 3A). Statistical Wilcoxon tests support this hypothesis (p-values of 0.25 for δ^13^C_16:0_ and 0.75 for δ^13^C_18:0_, see ESM-1); combustion temperature is the main factor for grouping needle samples.

In the case of branch samples, dead samples (unburnt and burnt up to 350 ºC) are enriched in ^13^C by 4–6 ‰ relative to the fresh samples; at 450 ºC both samples recorded similar values. The enrichment in ^13^C in dead branches compared to fresh branches could be also related to the absence of bark tissue in dead branches, which are depleted in ^13^C (Jambrina-Enríquez et al. 2019) or to the decomposition of organic matter, which results in an enrichment of ^13^C in the residual matter (Novák et al. 2003). On the other hand, these ^13^C-enriched signatures are in the range of terrestrial animal fats (δ^13^C_16:0_ and δ^13^C_18:0_ values vary between ~  − 30‰ and − 25‰ for both fatty acids e.g., Craig et al. 2013; Lucquin et al. 2016) which is relevant for the interpretation of fatty acid isotope data from combustion-derived sediment of unknown origin. Unlike needle samples, fresh and dead branches δ^13^C values remained mostly stable up to a charring temperature of 350 ºC with a shift to higher δ^13^C values at 450 ºC (~ 4 ‰) (Fig. 2AB2). Similar findings on fresh branches samples charred up to 350 ºC were reported by Jambrina-Enríquez et al. (2019). δ^13^C_16:0_ and δ^13^C_18:0_ values of fresh and dead branches allow differentiation between fresh and dead samples charred up to 350 ºC but not among different combustion temperatures (*p*-value = 0.125, ESM-1, Fig. 3B). At higher temperatures (450 ºC), the state of the degradation cannot be discerned and the charred fresh sample falls in the area of unburnt and burnt dead branches (Fig. 3B). The Mann–Whitney test performed on the δ^13^C_16:0_ and δ^13^C_18:0_ values in the different anatomical parts (fresh and dead) of *P. canariensis* showed that fresh and dead needles burning under smouldering conditions (≤ 250 ºC) were different than all the charred branch samples (fresh and dead), and charred needles (fresh and dead) burnt at 350–450 ºC (*p* value < 0.0001 for δ^13^C_16:0_ and 0.001 for δ^13^C_18:0_, ESM-1). Resins were depleted in ^13^C with increasing temperature combustion by ~ 2‰, and the δ^13^C remained stable from 150 ºC to 350 ºC (Figs. 2AB3 and 3C). When we compared the δ^13^C_16:0_ and δ^13^C_18:0_ values between unburnt and burnt charred pine tissue and resin, resin were enriched in ^13^C by about 10 ‰ compared to needles and by ~ 4 ‰ compared to fresh branches (*p*-values = 0.004 for δ^13^C_16:0_ and 0.048 for δ^13^C_18:0,_ ESM-1), but did not differ from the dead branches samples (*p*-values = 0.317 for δ^13^C_16:0_ and 0.413 for δ^13^C_18:0_, ESM-1) and charred tissues at 450 ºC (*p*-values = 0.143 for δ^13^C_16:0_ and 0.371 for δ^13^C_18:0_, ESM-1). Comparative studies of bulk δ^13^C signatures on fresh branches and resin from conifers reported more ^13^C-enriched signatures in resins than in other anatomical parts from the same plant (by 2–4 ‰), which can be explained by the process of resin biosynthesis (Dal Corso et al. 2017). Similar to dead branches, the ^13^C-enriched signatures are in the range of terrestrial animal fats.

**Fig. 4:**
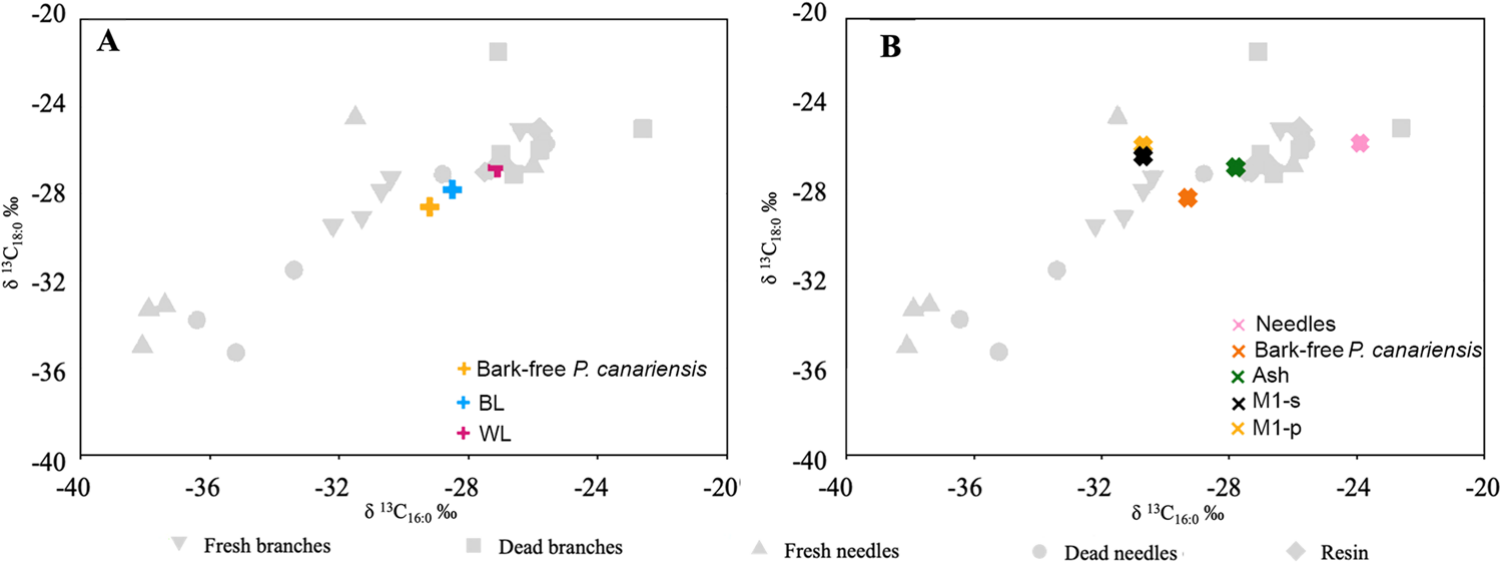
Plots of the δ^13^C_16:0_ and δ^13^C_18:0_ values of pine tissue from open fire experiments by Buonasera et al. (2019) (**A**) and pine wildfire (this study) (**B**). Detailed temperature data of reference samples is given in Fig. 3

We compared our experimental results under oxygen-limited conditions with published δ^13^C values of short-chain fatty acids from an open-air fire experiment fuelled with dried fresh and mostly bark-free *P. canariensis* (Buonasera et al. 2019). The δ^13^C signatures of the control sample (bark-free *P. canariensis)* plotted in the area of our fresh branch samples and corroborate the woody tissue source and the low state of degradation. The black layer sample plots in the area of our fresh branch samples charred up to 350 ºC and close to the dead needle δ^13^C values charred at 450 ºC (Fig. 4A). However, the δ^13^C values obtained from the black layer are similar to the δ^13^C values registered by fresh branches (*p*-values = 0.381 for δ^13^C_16:0_ and 0.857 for δ^13^C_18:0,_ ESM-1) and the subsurface temperature of the open-air fire was consistently < 427 ºC, and no needles were introduced into the fire (Buonasera et al. 2019). Therefore, the δ^13^C signatures of the black layer (subsurface substrate) seem to indicate a signature of charred fresh branches at combustion temperatures below 450 ºC, and not of charred needles at 450 ºC. The isotopic values of the ash layer (white layer), which sustained a surface combustion temperature > 600 ºC (Buonasera et al. 2019), plots in the area of our fresh branches charred at 450 ºC as well in the area of charred resin (Fig. 4A). Buonasera et al. (2019) did not report macroscopic evidence of resin residues. However, our results offer this possibility, which would not be surprising given the nature of the fuel.

The *P. canariensis* wildfire ash sample plots close to the δ^13^C values of the white layer from the open-air fire experiment of Buonasera et al. (2019) (~ 600 ºC) and close to our dead and fresh branch samples charred at 450 ºC corroborating the presence of high temperature heating (Fig. 4B). Like the open-fire experiment, the wildfire ash sample plots close to charred resins (Fig. 4B). The charred branch samples (bark-free pine) of the *P. canariensis* wildfire plots close to the control and black layer samples of Buonasera et al. (2019) and in the area of our experimentally heated fresh branch samples charred below 450 ºC, suggesting wildfire substrate combustion temperatures below 450ºC and fresh charred organic matter. In fact, the inner section of charred branches collected in the field remained unburnt. However, the wildfire charred needles plot close to our reference dead or fresh charred needles around 450 ºC (Fig. 4B). This divergence in the substrate combustion temperatures could be related to a higher degree of degradation/maturity of the needles covering the substrate than those in the reference sample. In this case, the enrichment in ^13^C could be related to microbial activity. In absence of charring processes, which protect organic matter from oxidation and microbial activity, several studies performed on fresh leaves, needles, and mosses and their top soils indicated an increase in long-chain fatty acids (*n*C_24:0_ to *n*C_30:0_) δ^13^C values by ~ 2–4 ‰ from fresh plant material in the humic horizon, mostly decomposed organic matter, but no further isotope fractionation from there downwards (Chikaraishi and Naraoka 2006; Griepentrog et al. 2015, 2016; Hirave et al. 2020).

Sediment samples collected below the ash layer (M1s: 0–5 cm deep and M1p: 5–10 cm deep below the surface) show similar δ^13^C values between them and plot in the area of fresh branches charred up to 350ºC and fresh or dead needles charred at 350 ºC (Fig. 4B). These temperatures at the surface (below 350 ºC) are consistent with maximum temperatures (370 °C at 2.5 cm, 156 °C at 5 cm, and 74 °C at 10 cm) recorded for wood-fuelled fires under different soil textures and soil moisture (Busse et al. 2010) and those recorded in the black layer (basalt substrate) at 3–5 cm deep below the surface (~ 300 ºC) by Buonasera et al. (2019), and agree with the absence of visible thermal alteration features (i.e., rubefaction) in the subsampled sediments.

**Fig. 5:**
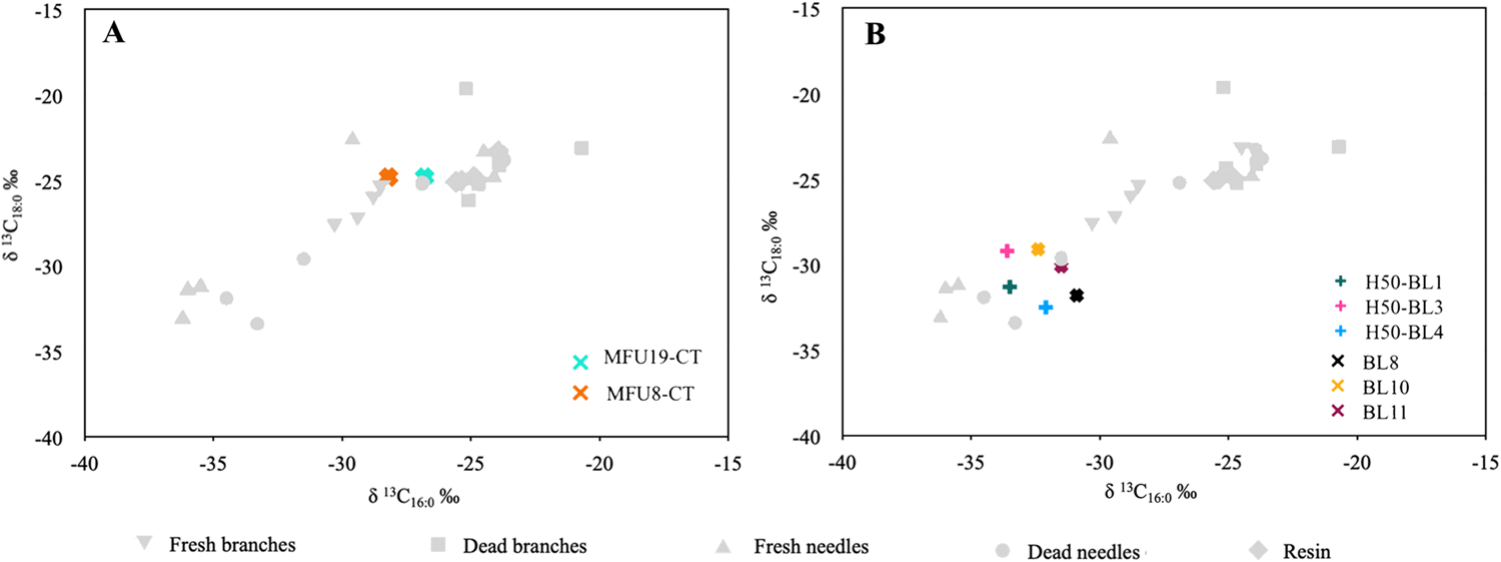
Plots of the δ^13^C_16:0_ and δ^13^C_18:0_ values of charred organic matter from combustion zone tramples (MFU8 and MFU19, Roques de García Rockshelter, Tomé et al 2022 (**A**) and hearth residues from El Salt (H50) and Crvena Stijena (SU XXIV: BL8,10,11) (Jambrina-Enríquez et al. 2019) (**B**). These are compared with modern fresh and dead pine reference samples (this study). The modern δ^13^C values were corrected by + 1.9‰ to match archaeological values. WL: white layer; BL: black layer, CT: Combustion residue Trample. Detailed temperature data for reference samples is given in Fig. 3

Comparison of our experimental and wildfire short-chain fatty acid δ^13^C values results with existing archaeological data from Roques de García (indigenous archaeological site, Tenerife), El Salt (Middle Palaeolithic, Spain) and Crvena Stijena (Middle Palaeolithic, Montenegro) required that the δ^13^C values of modern samples were adjusted for the addition of effects from post-industrial carbon or Suess effect (the correction factor was + 1.9‰, assuming a δ^13^C value at the time of sampling of − 8.3‰, Keeling et al. 2010 and a preindustrial atmospheric δ^13^C value of − 6.4‰, McCarroll and Loader 2004) (Fig. 5).

For Roques de García, the Canarian indigenous site, Tomé et al. (2022) report a mixture of juniper and other conifer sources in a combustion trample identified within the stratigraphic sequence (MFU 8) by comparison with δ^13^C_16:0_ and δ^13^C_18:0_ values of fresh juniper branches (Tomé et al. 2022) and fresh *P. Canariensis* branches (Jambrina-Enríquez et al. 2019). The corresponding δ^13^C_16:0_ and δ^13^C_18:0_ values from MFU 8 plot closes our experimentally charred fresh pine branches (combustion temperatures ≤ 350 ºC; Fig. 5A), pointing to the plausible presence of this taxa in the cited mix. The enrichment in ^13^C in our pine fresh branches references relative to those reported in fresh *P. Canariensis* branches by Jambrina-Enríquez et al. (2019) point to seasonality or canopy variations affecting the isotopic values, which has been observed in *n*-alkanes from angiosperms and conifers (Pedentchouk et al. 2008; Suh and Diefendorf 2018). In a different combustion trample from the same stratigraphic sequence, MFU 19, Tomé et al. (2022) report isotopic values falling within the fresh and dead branches area and close to the fresh needles charred at 350 ºC (Fig. 5A). No conifer biomarkers nor microscopic fragments of pine needles were observed, but a few microscopic conifer charcoal fragments were identified, possibly derived from the overlying white layer (Tomé et al. 2022). On the other hand, the sedimentary δ^13^C values in both combustion-derived occupation surfaces are lower than the those from the charred resin reference samples, suggesting low resin content.

Plots of the existing short-chain fatty acid δ^13^C values from the black layers (BL1, BL3 and BL4) of El Salt combustion structures (Jambrina-Enríquez et al. 2019) together with our experimental reference pine values suggest fresh or dead needles contribution and combustion temperatures ≤ 250 ºC. Plant sources reported by Leierer et al. (2019) and Jambrina-Enríquez et al. (2019) based on lipid biomarkers and compound-specific stable isotopes show a predominant angiosperm leafy content except for BL1, which contains angiosperm woody topsoil residues. The divergence of different anatomical parts (leaves/needles vs. branches) and our results regardless of combustion temperatures, may be related to phylogenetic aspects. *Celtis australis* (angiosperms) fresh branches (Jambrina-Enríquez et al. 2019) are depleted in ^13^C by about 3 ‰ compared to our fresh pine branches (conifers). This δ^13^C difference between phylogenetic groups has been reported in previous studies (e.g. Diefendorf et al. 2011). Similarly, the fatty acid δ^13^C data from the black layer samples at Crvena Stijena (Jambrina-Enríquez et al. 2019) plot close to charred needles ≤ 250 ºC (Fig. 5B). However, since the presence of *n*-alkyl nitriles, which are indicative of combustion temperatures around 300–350 ºC (Jambrina-Enríquez et al. 2019) in these black layers, we discard plant tissues charred at low temperatures and suggest a mixture of fresh leafy and woody tissues charred at 350ºC. Previous studies based on compound-specific stable isotopes reported that BL 8 represents terrestrial animal fats and BLs 10 and 11 charred fresh woody residues (angiosperms) (Jambrina-Enríquez et al. 2019).

Organic residues (charred and uncharred) in archaeological combustion sediments or archaeological artefacts may derive from plant (different anatomical plant parts) or animal sources, or mixtures of both. The characterization of δ^13^C_16:0_ and δ^13^C_18:0_ signatures of different types of pine tissue (lower δ^13^C values in needles and higher δ^13^C values in branches) and the effect of the state of organic matter on δ^13^C_16:0_ and δ^13^C_18:0_ values (lower δ^13^C values in fresh branches and higher δ^13^C values in dead branches) as well as the effect of thermal degradation (low: ≤ 250 ºC or high: ≥ 450ºC temperatures) reported in our reference dataset point to the use of C-specific isotope analysis of short-chain fatty acids as a complementary proxy to paleofire research to advance in the identification of the origin and the state of organic charred residues and combustion temperatures in archaeological black layers. Our study also highlights that some pine tissues and plant exudates such as resins, but also the mature state of the organic matter (e.g., dead branches) or the effect of higher combustion temperatures (450 ºC) may yield similar high δ^13^C values to those reported in animal fats. Hearth experimental studies showed that organic residues with animal fats are enriched in ^13^C (~ 4 ‰) compared to plant residues (dried fresh leafy and branches), and that low δ^13^C_16:0_ values (δ^13^C_16:0_ ≤  − 31‰) could indicate leaf oils and low contribution and preservation of terrestrial animal fat (Jambrina-Enríquez et al. 2019), with some exceptions as the higher δ^13^C_16:0_ and δ^13^C_18:0_ in some seeds (Steele et al. 2010). However, pine dead branches and resin, as well pine tissues charred at 450 ºC report δ^13^C_16:0_ and δ^13^C_18:0_ values in the range of terrestrial animal fats (δ^13^C_16:0_ and δ^13^C_18:0_ values vary between ~  − 30 and − 25 ‰ for both fatty acids (e.g., Craig et al. 2013; Lucquin et al. 2016). Moreover, phylogeny and seasonality affect the δ^13^C_16:0_ and δ^13^C_18:0_ values. The combination of stable isotopic and molecular approaches with other complementary analysis (e.g., micromorphology in combustion sediments) are required to obtain a more accurate source of the lipids (plant oils vs. animal fats).

## Conclusions

Our comparative study of short-chain fatty acid δ^13^C signatures from experimental pine samples (unheated and heated in controlled laboratory conditions), pine wildfire samples, experimental open-fire samples (Buonasera et al. 2019), and archaeological combustion structures and tramples (Jambrina-Enríquez et al. 2019; Tomé et al. 2022) highlight the preservation potential of lipid biomarkers and their isotopic fingerprints in archaeological black layers associated with combustion. We have shown that there are isotopic biomarkers for different anatomical parts of pine heated at different temperature ranges. We have also identified isotopic biomarkers for different preservation states of pine in unheated and heated pine samples. At smouldering conditions (≤ 250 ºC), pine needles and fresh and dead wood have distinct isotopic biomarkers. At higher temperatures, different anatomical parts converge and charred needles at 350 ºC overlap with charred fresh branches. Our observations have also advanced our understanding of different aspects of pyrotechnology in archaeological contexts. For Los Roques de García, we identified possible fresh pine input and low combustion temperatures (≤ 350 ºC) in the human occupation surfaces and in the BLs from El Salt (H50) and Crvena Stijena (SU XXIV) we identified a fresh state of the charred organic matter and low combustion temperatures (≤ 250 ºC).

Our results highlight the potential of short-chain fatty acid δ^13^C signatures to assess the state of plant input (fresh and dead) in natural or anthropogenic sediment and soil samples, with some limitations: i) needles charred at smouldering conditions (≤ 250 ºC) are more ^13^C-depleted than needles charred at 350 ºC and 450 ºC, but the state of the organic matter cannot be distinguished at this temperature range, ii) fresh branches charred up to 350 ºC are more ^13^C-depleted than dead branches, but at higher temperatures the effect of thermal degradation hampers the distinction between fresh and dead, iii) needles charred at smouldering conditions (≤ 250 ºC) are more ^13^C-depleted than branches, but these become indistinguishable at higher temperature, and iv) resins are more ^13^C-enriched than needles charred at smouldering conditions and fresh branches wood, but similar to dead branches wood. On the other hand, we indicated that the mature state of the organic matter, higher combustion temperatures and some plant exudates (resins) overlap in their δ^13^C signatures with terrestrial animal fats. Moreover, phylogenetic groups and seasonality may affect δ^13^C signatures.

Future research on *n*C_16:0_ and *n*C_18:0_ fatty acid isotope analysis among major plant groups (angiosperm and conifers) covering different anatomical parts, (branches leaves/needles, resin), state of degradations (driftwood, fresh and dead) and degree of combustion (charred and uncharred, low and high temperatures) is necessary to expand the reference dataset. We recommend a combination of compound-specific isotopic analysis and molecular (e.g., alkanes, aromatics, terpenoids, *n*-alkyl nitriles) analysis to approach possible sources and preservation states of organic matter.

## Supplementary Information

Below is the link to the electronic supplementary material.

Supplementary file1 (PDF 424 KB)

## Data Availability

All relevant data are within the manuscript and supplementary material.

## References

[CR1] Aldeias V (2017). Experimental Approaches to Archaeological Fire Features and Their Behavioral Relevance. Curr Anthropol.

[CR2] Aldeias V, Dibble HL, Sandgathe D, Goldberg P, McPherron SJ (2016). How heat alters underlying deposits and implications for archaeological fire features: a controlled experiment. J Archaeol Sci.

[CR3] Allue E, Mas B (2020). The meaning of Pinus sylvestris-type charcoal taphonomic markers in Palaeolithic sites in NE Iberia. J Archaeol Sci Rep.

[CR4] Ballentine DC, Macko SA, Turekian VC (1998). Variability of stable carbon isotopic compositions in individual fatty acids from combustion of C4 and C3 plants: implications for biomass burning. Chem Geol.

[CR5] Braadbaart F, Poole I (2008). Morphological, chemical and physical changes during charcoalification of wood and its relevance to archaeological contexts. J Archaeol Sci.

[CR6] Brito ADCV, Rodríguez RG (2008). Hornos de brea en Tenerife: Identificación y catalogación. Revista De Historia Canaria.

[CR7] Buonasera TY, Tremayne AH, Darwent CM, Eerkens JW, Mason OK (2015). Lipid biomarkers and compound specific δ^13^C analysis indicate early development of a dual-economic system for the Arctic Small Tool tradition in northern Alaska. J Archaeol Sci.

[CR8] Buonasera T, Herrera-Herrera AV, Mallol C (2019). Experimentally derived sedimentary, molecular, and isotopic characteristics of bone-fueled hearths. J Archaeol Method Theory.

[CR9] Busse MD, Shestak CJ, Hubbert KR, Knapp EE (2010). Soil physical properties regulate lethal heating during burning of woody residues. Soil Sci Soc Am J.

[CR10] Campbell GS, JrJD J, Bristow KL, Hungerford RD (1995). Soil temperature and water content beneath a surface fire. Soil Sci.

[CR11] Chikaraishi Y, Naraoka H (2006). Carbon and hydrogen isotope variation of plant biomarkers in a plant–soil system. Chem Geol.

[CR12] Chikaraishi Y, Naraoka H, Poulson SR (2004). Carbon and hydrogen isotopic fractionation during lipid biosynthesis in a higher plant (Cryptomeria japonica). Phytochemistry.

[CR13] Chikaraishi Y, Naraoka H, Poulson SR (2004). Hydrogen and carbon isotopic fractionations of lipid biosynthesis among terrestrial (C3, C4 and CAM) and aquatic plants. Phytochemistry.

[CR14] Chil Naranjo G (1990) Anatomía patológica de los aborígenes canarios (I) lesiones de huesos. 8(82–93):43–44

[CR15] Choy K, Potter BA, McKinney HJ, Reuther JD, Wang SW, Wooller MJ (2016). Chemical profiling of ancient hearths reveals recurrent salmon use in Ice Age Beringia. Proc Natl Acad Sci.

[CR16] Climent J, Gil L, Pardos JA (1998). Xylem anatomical traits related to resinous heartwood formation in Pinus canariensis Sm. Trees.

[CR17] Craig OE, Saul H, Lucquin A, Nishida Y, Taché K, Clarke L, ... Jordan P (2013) Earliest evidence for the use of pottery. Nature, 496(7445): 351–354 10.1038/nature12109 23575637

[CR18] Dal Corso J, Schmidt AR, Seyfullah LJ (2017). Evaluating the use of amber in palaeoatmospheric reconstructions: The carbon-isotope variability of modern and Cretaceous conifer resins. Geochim Cosmochim Acta.

[CR19] Diefendorf AF, Mueller KE, Wing SL, Koch PL, Freeman KH (2010). Global patterns in leaf ^13^C discrimination and implications for studies of past and future climate. Proc Natl Acad Sci.

[CR20] Diefendorf AF, Freeman KH, Wing SL, Graham HV (2011). Production of *n*-alkyl lipids in living plants and implications for the geologic past. Geochim Cosmochim Acta.

[CR21] Diefendorf AF, Sberna DT, Taylor DW (2015). Effect of thermal maturation on plant-derived terpenoids and leaf wax *n*-alkyl components. Org Geochem.

[CR22] Galván B, Hernández CM, Mallol C, Mercier N, Sistiaga A, Soler V (2014). New evidence of early Neanderthal disappearance in the Iberian Peninsula. J Hum Evol.

[CR23] Gamarra B, Kahmen A (2015). Concentrations and δ^2^H values of cuticular *n*-alkanes vary significantly among plant organs, species and habitats in grasses from an alpine and a temperate European grassland. Oecologia.

[CR24] Goodman KJ, Brenna JT (1992). High sensitivity tracer detection using high-precision gas chromatography-combustion isotope ratio mass spectrometry and highly enriched uniformly carbon-13 labeled precursors. Anal Chem.

[CR25] Griepentrog M, Eglinton TI, Hagedorn F, Schmidt MW, Wiesenberg GL (2015). Interactive effects of elevated CO_2_ and nitrogen deposition on fatty acid molecular and isotope composition of above-and belowground tree biomass and forest soil fractions. Glob Change Biol.

[CR26] Griepentrog M, Bodé S, Boeckx P, Wiesenberg GL (2016). The fate of plant wax lipids in a model forest ecosystem under elevated CO_2_ concentration and increased nitrogen deposition. Org Geochem.

[CR27] Henry A, Théry-Parisot I (2014). From Evenk campfires to prehistoric hearths: charcoal analysis as a tool for identifying the use of rotten wood as fuel. J Archaeol Sci.

[CR28] Hirave P, Wiesenberg GL, Birkholz A, Alewell C (2020). Understanding the effects of early degradation on isotopic tracers: implications for sediment source attribution using compound-specific isotope analysis (CSIA). Biogeosciences.

[CR29] Jambrina-Enríquez M, Herrera-Herrera AV, Mallol C (2018). Wax lipids in fresh and charred anatomical parts of the Celtis australis tree: Insights on paleofire interpretation. Org Geochem.

[CR30] Jambrina-Enríquez M, Herrera-Herrera AV, de Vera CR, Leierer L, Connolly R, Mallol C (2019) *n*-Alkyl nitriles and compound-specific carbon isotope analysis of lipid combustion residues from Neanderthal and experimental hearths: identifying sources of organic compounds and combustion temperatures. Quat Sci Rev 222:105899

[CR31] Keeling RF, Piper SC, Bollenbacher AF, Walker SJ (2010) Monthly atmospheric ^13^C/^12^C isotopic ratios for 11 SIO stations. Trends: a compendium of data on global change. United States. 10.3334/cdiac/atg.025

[CR32] Knicker H, Hilscher A, De la Rosa J, González-Pérez J, González-Vila FJ (2013). Modification of biomarkers in pyrogenic organic matter during the initial phase of charcoal biodegradation in soils. Geoderma.

[CR33] Kuo LJ, Herbert BE, Louchouarn P (2008). Can levoglucosan be used to characterize and quantify char/charcoal black carbon in environmental media?. Org Geochem.

[CR34] Leierer L, Jambrina-Enríquez M, Herrera-Herrera AV, Connolly R, Hernández CM, Galván B, Mallol C (2019). Insights into the timing, intensity and natural setting of Neanderthal occupation from the geoarchaeological study of combustion structures: A micromorphological and biomarker investigation of El Salt, unit Xb, Alcoy, Spain. Plos One.

[CR35] Leroi-Gourhan A (1973). Séminaire sur les structures d’habitat: témoins de combustión.

[CR36] Liu F, Ma M, Li G (2022). Prehistoric firewood gathering on the northeast Tibetan plateau: environmental and cultural determinism. Veg Hist Archaeobotany.

[CR37] Lucquin A, Gibbs K, Uchiyama J, Saul H, Ajimoto M, Eley Y, ... Craig OE (2016) Ancient lipids document continuity in the use of early hunter–gatherer pottery through 9,000 years of Japanese prehistory. Proc Natl Acad Sci 113(15):3991–3996 10.1073/pnas.1522908113 PMC4839459 27001829

[CR38] Mallol C, Hernández CM, Cabanes D, Sistiaga A, Machado J, Rodríguez A, Pérez L, Galván B (2013). The black layer of Middle Palaeolithic combustion structures. Interpretation and archaeostratigraphic implications. J Archaeol Sci.

[CR39] Mallol C, Mentzer S, Miller C, Nicosia C, Stoops G (2017). Combustion features. Archaeological Soil and Sediment Micromorphology.

[CR40] March RJ, Lucquin A, Joly D, Ferreri JC, Muhieddine M (2014). Processes of formation and alteration of archaeological fire structures: complexity viewed in the light of experimental approaches. J Archaeol Method Theory.

[CR41] March R (2013) Searching for the functions of fire structures in Eynan (Mallaha) and their formation processes: a geochemical approach. In: Bar-Yosef O, Valla FR (eds) Natufian Foragers in the Levant Terminal Pleistocene - Social Changes in Western Asia. International Monographs in Prehistory Archaeological Series. Ann Arbor, Michigan U.S.A., 19(17):227–284

[CR42] McCarroll D, Loader NJ (2004). Stable isotopes in tree rings. Quat Sci Rev.

[CR43] McKern HHG (1965). Volatile oils and plant taxonomy. J Proc R Soc NSW.

[CR44] Morley MW, Whallon R (2017). The Geoarchaeology of Crvena Stijena Site Formation Processes, Palaeoenvironments and Hominin Activity. Crvena Stijena in Cultural and Ecological Context Multidisciplinary Archaeological Research in Montenegro.

[CR45] Naranjo GC (1900). Anatomía patológica de los aborígenes canarios (I): lesiones de huesos. El Museo Canario.

[CR46] Novák M, Buzek F, Harrison AF, Přechová E, Jačková I, Fottová D (2003). Similarity between C, N and S stable isotope profiles in European spruce forest soils: implications for the use of δ^34^S as a tracer. App Geochem.

[CR47] Oros DR, Simoneit BR (2001). Identification and emission factors of molecular tracers in organic aerosols from biomass burning Part 1. Temperate Climate Conifers. App Geochem.

[CR48] Otto A, Simoneit BR, Wilde V (2007). Terpenoids as chemosystematic markers in selected fossil and extant species of pine (Pinus, Pinaceae). Bot J Linn Soc.

[CR49] Pedentchouk N, Sumner W, Tipple B, Pagani M (2008). δ^13^C and δD compositions of *n*-alkanes from modern angiosperms and conifers: an experimental set up in central Washington State. USA Org Geochem.

[CR50] Rodríguez de Vera C, Herrera-Herrera AV, Jambrina-Enríquez M (2020). Micro-contextual identification of archaeological lipid biomarkers using resin-impregnated sediment slabs. Sci Rep.

[CR51] Roussis V, Petrakis PV, Ortiz A, Mazomenos BE (1995). Volatile constituents of needles of five Pinus species grown in Greece. Phytochemistry.

[CR52] Shaw D (2017) Archaeobotanical Results from Crvena Stijena. In: Robert Whallon (ed) Crvena Stijena in Cultural and Ecological Context Multidisciplinary Archaeological Research in Montenegro. Podgorica: National Museum of Montenegro, Montenegrin Academy of Sciences and Arts, Special editions (Monographies and Studies), pp 307–340

[CR53] Spangenberg JE, Ogrinc N (2001). Authentication of vegetable oils by bulk and molecular carbon isotope analyses with emphasis on olive oil and pumpkin seed oil. Agric Food Chem.

[CR54] Steele VJ, Stern B, Stott AW (2010). Olive oil or lard?: distinguishing plant oils from animal fats in the archaeological record of the eastern Mediterranean using gas chromatography/combustion/isotope ratio mass spectrometry. Rapid Commun Mass Spectrom.

[CR55] Suh YJ, Diefendorf AF (2018). Seasonal and canopy height variation in *n*-alkanes and their carbon isotopes in a temperate forest. Org Geochem.

[CR56] Théry-Parisot I, Henry A (2012). Seasoned or green? Radial cracks analysis as a method for identifying the use of green wood as fuel in archaeological charcoal. J Archaeol Sci.

[CR57] Théry-Parisot I, Chabal L, Chrzavzez J (2010). Anthracology and taphonomy, from wood gathering to charcoal analysis. A review of the taphonomic processes modifying charcoal assemblages, in archaeological contexts. Palaeogeogr Palaeoclimatol Palaeoecol.

[CR58] Tomé L, Jambrina-Enríquez M, Égüez N, Herrera-Herrera AV, Davara J, Marrero Salas E, Arnay de la Rosa M, Mallol C (2022). Fuel sources, natural vegetation and subsistence at a high-altitude aboriginal settlement in Tenerife, Canary Islands: Microcontextual geoarchaeological data from Roques de García Rockshelter. Archaeol Anthropol Sci.

[CR59] Ustun O, Sezik E, Kurkcuoglu M, Baser KHC (2006). Study of the essential oil composition of Pinus sylvestris from Turkey. Chem Nat Compd.

[CR60] Vidal-Matutano P, Henry A, Théry-Parisot I (2017). Dead wood gathering among Neanderthal groups: charcoal evidence from Abric del Pastor and El Salt (Eastern Iberia). J Archaeol Sci.

[CR61] Vidal-Matutano P, Henry A, Carré A, Orange F, Théry-Parisot I (2021). Prehispanic fuel management in the Canary Islands: A new experimental dataset for interpreting Pinus canariensis micromorphological degradation patterns on archeological charcoal. Rev Palaeobot Palynol.

[CR62] Wiedemeier DB, Brodowski S, Wiesenberg GL (2015). Pyrogenic molecular markers: Linking PAH with BPCA analysis. Chemosphere.

[CR63] Wiesenberg GL, Lehndorff E, Schwark L (2009). Thermal degradation of rye and maize straw: lipid pattern changes as a function of temperature. Org Geochem.

[CR64] Woodbury SE, Evershed RP, Rossell JB (1998). δ^13^C analyses of vegetable oil fatty acid components, determined by gas chromatography-combustion-isotope ratio mass spectrometry, after saponification or regiospecific hydrolysis. J Chromatogr A.

