# Supplementary material for: Compound-specific carbon isotope analysis of short-chain fatty acids from Pine tissues: characterizing paleo-fire residues and plant exudates
Source: Archaeol Anthropol Sci. 2023 Jul 11;15(8):114. doi: 10.1007/s12520-023-01815-3 (PMC10333141; doi:10.1007/s12520-023-01815-3)
Supplement: Supplementary file 1 — Supplementary file1 (PDF 424 KB) [file 12520_2023_1815_MOESM1_ESM.pdf]

Compound-specific carbon isotope analysis of short-chain fatty acids from Pine tissues: characterizing paleo-fire residues and plant exudates

Margarita Jambrina-Enríquez[1,2*], Caterina Rodríguez de Vera[2], Javier Davara[2], Antonio V. Herrera-Herrera[2], Carolina Mallol[2,3,4]

[1] Departamento de Biología Animal, Edafología y Geología, Facultad de Ciencias, Universidad de La Laguna, San Cristóbal de La Laguna, Tenerife, Spain

[2] Archaeological Micromorphology and Biomarker Research Lab (AMBI Lab), Instituto Universitario de Bio-Orgánica Antonio González, Universidad de La Laguna, San Cristóbal de La Laguna, Tenerife, Spain

[3] Departamento de Geografía e Historia, Facultad de Humanidades, Universidad de La Laguna, San Cristóbal de La Laguna, Tenerife, Spain

[4] ICArEHB - Interdisciplinary Center for Archaeology and the Evolution of Human Behaviour, Universidade do Algarve, Faro, Portugal

***corresponding author: mjambrin@ull.edu.es**

Supplementary Information 1 (ESM-1) Results of parametric and non-parametric tests applied to compound-specific carbon isotope analysis of $C_{16:0}$ and $C_{18:0}$ fatty acids. H0 is assumed when p-value > α

Pearson's correlation coefficient			
**Fresh needles**			
Variables	Combustion temperature	$\delta^{13}C_{16:0}$	$\delta^{13}C_{18:0}$
Combustion temperature	1	0.916	0.670
$\delta^{13}C_{16:0}$	0.916	1	0.668
$\delta^{13}C_{18:0}$	0.670	0.668	1
**Dead needles**			
Variables	Combustion temperature	$\delta^{13}C_{16:0}$	$\delta^{13}C_{18:0}$
Combustion temperature	1	0.976	0.963
$\delta^{13}C_{16:0}$	0.976	1	0.938
$\delta^{13}C_{18:0}$	0.963	0.938	1
**Fresh branches**			
Variables	Combustion temperature	$\delta^{13}C_{16:0}$	$\delta^{13}C_{18:0}$
Combustion temperature	1	0.853	0.929
$\delta^{13}C_{16:0}$	0.853	1	0.942
$\delta^{13}C_{18:0}$	0.929	0.942	1
**Dead branches**			
Variables	Combustion temperature	$\delta^{13}C_{16:0}$	$\delta^{13}C_{18:0}$
Combustion temperature	1	0.193	0.455
$\delta^{13}C_{16:0}$	0.193	1	0.730
$\delta^{13}C_{18:0}$	0.455	0.730	1

Wilcoxon test(**α=0.05**)				
Parameter	V	Expected Value	Variance	p-value (bilateral)
H0: The state of the degradation in needles samples (fresh or dead) cannot be differentiated by $\delta^{13}C$ values				
$\delta^{13}C_{16:0}$	0	3.000	3.500	0.25
$\delta^{13}C_{18:0}$	3	3.000	3.500	0.75
H0: $\delta^{13}C$ values of fresh and dead branches do not allow differentiation between fresh samples and dead samples charred up to 350 ºC				
$\delta^{13}C_{16:0}$	0	5.000	7.500	0.125
$\delta^{13}C_{18:0}$	0	5.000	7.500	0.125

Mann-Whitney test (**α=0.05**)					
Parameter	U	U (standardized)	Expected value	Var (U)	p-value
H0: Fresh and dead needles burnt at smoldering temperatures (≤250ºC) does not have differences in $\delta^{13}C$ values with those obtained in other samples (branches and needles burnt at 350ºC and 450ºC)					
$\delta^{13}C_{16:0}$	0	0.000	39.000	130.000	<0.0001
$\delta^{13}C_{18:0}$	0	-3.378	39.000	129.886	0.001
H0: Resin $\delta^{13}C$ values are similar than $\delta^{13}C$ values obtained in dead branches					
$\delta^{13}C_{16:0}$	14.500	0.000	10.000	16.528	0.317
$\delta^{13}C_{18:0}$	14.000	0.000	10.000	16.667	0.413
H0: Resin $\delta^{13}C$ values are similar than $\delta^{13}C$ values obtained in needles and fresh branches					
$\delta^{13}C_{16:0}$	0	0.000	16.000	34.667	0.004
$\delta^{13}C_{18:0}$	4	0.000	16.000	34.667	0.048
H0: Resin $\delta^{13}C$ values are similar than $\delta^{13}C$ values obtained in charred material burnt at 450ºC					
$\delta^{13}C_{16:0}$	13.500	0.000	8.000	11.857	0.143
$\delta^{13}C_{18:0}$	11.500	0.000	8.000	11.857	0.371
H0: The $\delta^{13}C$ values obtained from a BL of an open fire made with pine wood are similar to the $\delta^{13}C$values registered by fresh branches					
$\delta^{13}C_{16:0}$	2	0.000	5.000	6.667	0.381
$\delta^{13}C_{18:0}$	5	0.000	5.000	6.667	0.857
